# Zebrafish as a Model for Neurological Disorders

**DOI:** 10.3390/ijms23084321

**Published:** 2022-04-13

**Authors:** Nadia Soussi-Yanicostas

**Affiliations:** NeuroDiderot, Inserm, Université Paris Cité, F-75019 Paris, France; nadia.soussi@inserm.fr

Over the past two decades, the simplicity and the versatility of the zebrafish (*Danio rerio*) have helped make it one of the main animal models used to address an increasing number of issues, from fundamental research to clinical investigations, drug discovery [1] and applied toxicology [2]. Its small size, rapid external development, almost full transparency, and the abundant tools available to study it, whether molecular (fluorescent calcium sensors, optogenetics, etc.) or genetic (transgenesis, gene editing, etc.), have made the zebrafish embryo an unparalleled model for in vivo investigations in the neurosciences [3]. Its central nervous system displays the characteristic organization found in vertebrates: a four-lobed brain, a spinal cord, and a neural crest; the biochemistry underlying the functioning of neuronal networks is also closely similar. All neurotransmitters (glutamate, γ-aminobutyric acid (GABA), etc.) and neuromodulators (oxytocin, somatostatin, etc.), and their receptors, and all neuron types (dopaminergic, cholinergic, etc.) and glial cell types (oligodendrocytes, microglia, etc.), exhibit full evolutionary conservation between the fish and mammals [4]. The zebrafish model and the many advantages it offers have already been thoroughly reviewed elsewhere, and so will not be described further here. The usefulness of the zebrafish and its relevance as an in vivo model in the neurosciences are plainly evident in the research articles and reviews that make up this special issue of International Journal of Molecular Sciences “Zebrafish as a model for neurological disorders”. 

In an illustration of the simplicity and power of the zebrafish as a genetic tool, two studies have made use of the relatively new CRISPR/Cas9 technology of gene editing to produce loss-of-function alleles of genes of interest prior to analyzing the induced phenotypes. Starting from a consanguineous family of whom several members presented macrocephaly, facial hypoplasia, hypotonia, and intellectual disability, Confino et al. [5] identified the causative mutation in the *FBXL3* gene encoding F-box and leucine-rich repeat protein 3 and then generated a loss-of-function allele of *fbxl3a*, one of the two zebrafish paralogs of this gene. Interestingly, the results suggested that in contrast to the human gene, *fbxl3a* is not essential for brain development, but is a key component of the circadian clock, as is the case in mice. Using the same technology, Rodriguez-Ortiz and Martinez-Torres [6] produced a loss-of-function mutant of *hcn2b*, one of the two zebrafish paralogs of the *HCN2* gene encoding a hyperpolarization-activated cyclic nucleotide gated (HCN) channel. This gene has been found mutated in humans presenting generalized epilepsy, and the zebrafish *hcn2b* mutant exhibits epileptiform seizures and epilepsy-related behaviors, highlighting the strong evolutionary conservation of the neurochemical processes underlying neuron excitation.

In an example of the broad variety of research made possible by this small fish, Chen et al. [7] used the adult zebrafish as a model to study the effects of tempeh, a traditional fermented soybean food product consumed mainly in Indonesia, on gut microbiota and anxiety. The interest in tempeh stems from the large amount of GABA it contains, and its widely alleged beneficial effects on anxiety, insomnia, hypertension, and diabetes. Interestingly, after two weeks of feeding with tempeh-containing food, adult zebrafish showed markedly decreased anxiety and marked changes in the composition of their microbiota, with a decrease in potentially pathogenic Proteobacteria and an increase in beneficial *Bifidobacterium* probiotics. In addition, qRT-PCR showed that tempeh triggered an increased accumulation of *bdnf* and several transcripts involved in serotonin synthesis (*tph1b* and *tph2*), demonstrating an evolutionarily conserved beneficial and anxiolytic effect of tempeh. 

Three articles highlight the usefulness of the zebrafish as a model for addressing fundamental but still poorly understood issues in the neurosciences, such as the functions of glial cells, adult neurogenic niches, or the processes controlling axon growth and guidance. Ceriani and Whitlock 2021 [8] used classical immunocytochemical methods to address a key issue in the neurosciences, namely the ability of the adult brain to generate new neurons in response to metabolic changes, with a focus on the hypothalamus, a well-known neurogenic region in the mammalian brain. This study first showed that gonadotropin-releasing hormone (GnRH), but not testosterone, triggered neurogenesis in the adult hypothalamus. Moreover, the cross-reactivity of several antibodies directed against mammalian proteins also enabled the precise characterization of the neurogenic cells in the preoptic region of the adult zebrafish hypothalamus, highlighting the strong similarities, but also the differences in the neurogenic niches between mammals and fish. Pereida-Jaramillo et al. [9] used transgenic fluorescent calcium sensors to visualize in vivo the transient calcium uptakes underlying the depolarization of neurons, and the membrane currents of glial cells whose function remains poorly understood. First, a calcium sensor expressed under the control of the glial *gfap* promoter was instrumental in demonstrating for the first time that rostro-caudal calcium waves occur in cerebellar Bergmann glial cells starting from 5 dpf. Moreover, using heptanol, a well-known gap junction blocker, the authors showed that calcium wave propagation, but not initiation, required fully functional gap junctions. Knickmeyer et al. [10] investigated the role of bone morphogenetic protein (BMP) signaling, especially BMP4, in optic nerve pathfinding using a transgenic line encoding the zebrafish *bmp4* gene under the heat-inducible *hsp70* promoter, allowing precise overexpression timing. The authors thereby showed that BMP signaling was dynamic and overexpression of *bmp4* impaired both the elongation of optic nerves and their crossing at the optic chiasm, suggesting for the first time a requirement for BMP signaling in retinal ganglion cell projections to the brain. 

The article of Park et al. [11] illustrates the relevance of the zebrafish embryo as a model for toxicological studies, including neurotoxicology. In their study, the authors used zebrafish embryos to investigate both the developmental toxicity and the neurotoxicity of acrylamide, a multipurpose chemical used in large quantities worldwide. This study provides evidence that acrylamide is toxic to zebrafish embryos, causing both developmental (teratogenic) defects and neurotoxicity, as shown by motor deficits and microcephaly in acrylamide-exposed individuals. 

This special issue of International Journal of Molecular Sciences also includes two reviews highlighting the usefulness and relevance of the zebrafish as a model in the clinical neurosciences. Barbereau et al. [12] offer a comprehensive review showing how transgenic lines expressing pathogenic variants of the tau protein found in human patients presenting various tauopathies, such as frontotemporal dementia (FTD), progressive supranuclear palsy (PSP), or Pick’s disease, have improved our knowledge of these disorders and enabled advances in various therapeutic approaches. Finally, in their review on axon-glia interactions during axon regeneration, Gonzalez and Allende [13] describe the novel tools that have taken forward our understanding of axon regeneration and its dynamics in the peripheric nervous system, such as advanced microscopy techniques and laser-mediated injury. 
